# Chlorido[1-diphenyl­phosphanyl-3-(phenyl­sulfan­yl)propane-κ^2^ *P*,*S*](η^5^-penta­methyl­cyclo­penta­dien­yl)iridium(III) chloride monohydrate

**DOI:** 10.1107/S1600536812021964

**Published:** 2012-05-31

**Authors:** Gerd Ludwig, Marcus Korb, Tobias Rüffer, Heinrich Lang, Dirk Steinborn

**Affiliations:** a Institut für Chemie – Anorganische Chemie, Martin-Luther-Universität Halle-Wittenberg, D-06120 Halle, Kurt-Mothes-Strasse 2, Germany; b Institut für Chemie – Anorganische Chemie, Technische Universät Chemnitz, D-09111 Chemnitz, Strasse der Nationen 62, Germany

## Abstract

The crystal structure of the title compound, [Ir(C_10_H_15_)Cl(C_21_H_21_PS)]Cl·H_2_O, consists of discrete [Ir(η^5^-C_5_Me_5_)Cl{Ph_2_P(CH_2_)_3_SPh-κ*P*,κ*S*}]^+^ cations, chloride anions and water mol­ecules. The Ir^III^ atom is coordinated by an η^5^-C_5_Me_5_ ligand, a chloride and a Ph_2_P(CH_2_)_3_SPh-κ*P*,κ*S* ligand, leading to a three-legged piano-stool geometry. In the crystal, two water molecules and two chloride anions are linked by weak O—H⋯Cl hydrogen bonding into tetra­mers that are located on centers of inversion. The H atoms of one of the methyl groups are disordered and were refined using a split model.

## Related literature

Analogous iridium(III) complexes with Ph_2_PCH_2_SPh ligands have been described by Valderrama *et al.* (1997) ▶. For arene ruthenium(II) complexes having ω-diphenyl­phosphanyl-functionalized alkyl phenyl sulfide ligands Ph_2_P(CH_2_)_*n*_SPh (*n* = 1–3), see: Ludwig *et al.* (2012 ▶). For an overview of the strength of hydrogen bonds, see: Steiner (2002 ▶).
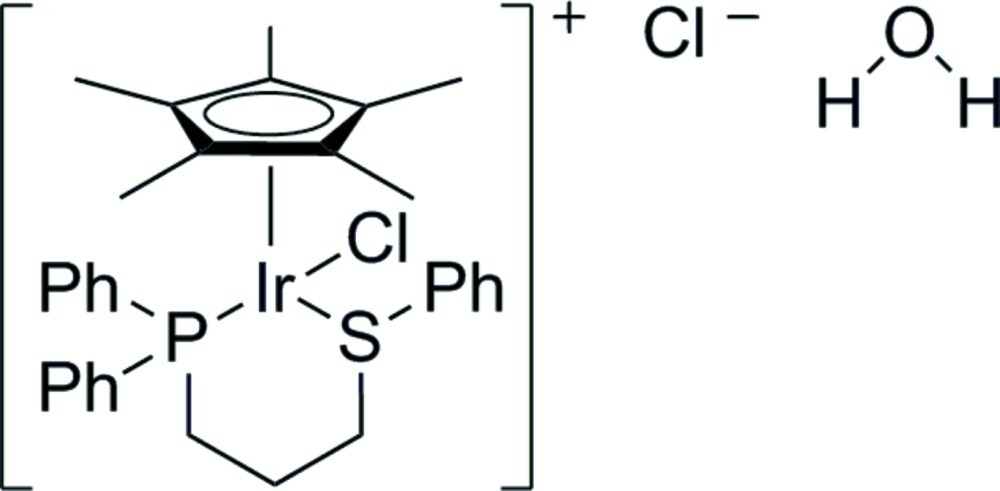


## Experimental

### 

#### Crystal data

[Ir(C_10_H_15_)Cl(C_21_H_21_PS)]Cl·H_2_O
*M* *_r_* = 752.74
Monoclinic, 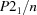

*a* = 11.0720 (3) Å
*b* = 8.9617 (2) Å
*c* = 30.6266 (7) Å
β = 100.000 (2)°
*V* = 2992.72 (13) Å^3^
*Z* = 4
Mo *K*α radiation
μ = 4.79 mm^−1^
*T* = 100 K
0.25 × 0.20 × 0.20 mm

#### Data collection

Oxford Diffraction Gemini S diffractometer
Absorption correction: multi-scan (*CrysAlis PRO*; Oxford Diffraction, 2006 ▶) *T*_min_ = 0.638, *T*_max_ = 1.000
27405 measured reflections
5513 independent reflections
5098 reflections with *I* > 2σ(*I*)
*R*_int_ = 0.047

#### Refinement

*R*[*F* ^2^ > 2σ(*F* ^2^)] = 0.041
*wR*(*F* ^2^) = 0.088
*S* = 1.25
5513 reflections
337 parameters
147 restraints
H atoms treated by a mixture of independent and constrained refinement
Δρ_max_ = 1.68 e Å^−3^
Δρ_min_ = −1.50 e Å^−3^

### 

Data collection: *CrysAlis PRO* (Oxford Diffraction, 2006 ▶); cell refinement: *CrysAlis PRO*; data reduction: *CrysAlis PRO*; program(s) used to solve structure: *SHELXS97* (Sheldrick, 2008 ▶); program(s) used to refine structure: *SHELXL97* (Sheldrick, 2008 ▶); molecular graphics: *DIAMOND* (Brandenburg, 2001 ▶); software used to prepare material for publication: *SHELXL97*.

## Supplementary Material

Crystal structure: contains datablock(s) I, global. DOI: 10.1107/S1600536812021964/nc2278sup1.cif

Structure factors: contains datablock(s) I. DOI: 10.1107/S1600536812021964/nc2278Isup2.hkl

Additional supplementary materials:  crystallographic information; 3D view; checkCIF report

## Figures and Tables

**Table 1:** Hydrogen-bond geometry (Å, °)

*D*—H⋯*A*	*D*—H	H⋯*A*	*D*⋯*A*	*D*—H⋯*A*
O1—H1*O*⋯Cl2	0.95 (6)	2.27 (6)	3.216 (6)	177 (10)
O1—H2*O*⋯Cl2^i^	0.96 (8)	2.26 (11)	3.196 (6)	165 (13)

Symmetry code: (i) 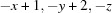.

**Fig. 1.**
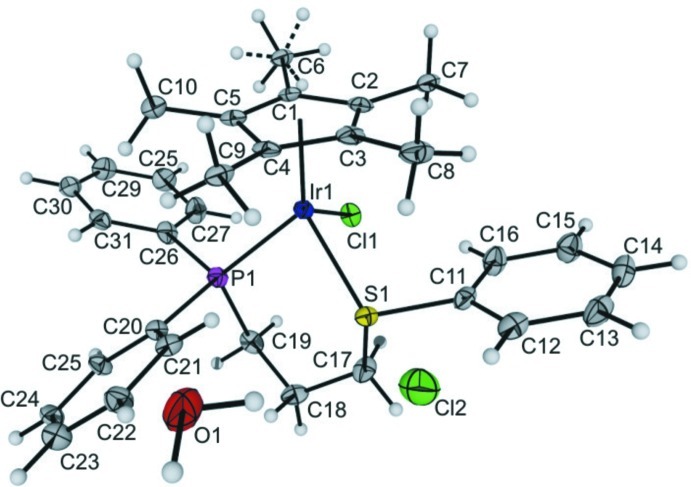
Structure of title compound [Ir(η5-C5Me5)Cl{Ph2P(CH2)3SPh-κP,κS}]Cl.H2O. Displacement ellipsoids are drawn at the 50% probability level and the H atoms are shown as small spheres of arbitrary radii. Disordering is shown as full and dashed bonds.

## supplementary crystallographic information

### Comment

Reaction of the ω-diphenylphosphanyl-functionalized alkyl phenyl sulfide Ph_2_P(CH_2_)_3_SPh with the dinuclear chlorido-bridged iridium complex [{Ir(η^5^-C_5_Me_5_)Cl_2_}_2_] afforded the title compound [Ir(η^5^-C_5_Me_5_)Cl{Ph_2_P(CH_2_)_3_SPh-κ*P*,κ*S*}]Cl^.^H_2_O. Similar iridium(III) and ruthenium(II) complexes with ω-diphenylphosphanyl-functionalized alkyl phenyl sulfide ligands Ph_2_P(CH_2_)*_n_*SPh (*n* = 1–3) have been reported by Valderrama (1997) and Ludwig (2012). Crystals of the title compound are built up from [Ir(η^5^-C_5_Me_5_)Cl{Ph_2_P(CH_2_)_3_SPh-κ*P*,κ*S*}]^+^ cations, chloride anions and water molecules. The iridium atom is slightly distorted octahedrally coordinated by a η^5^-C_5_Me_5_, a chlorido, and a chelating Ph_2_P(CH_2_)_3_SPh-κ*P*,κ*S* ligand, which leads to a three-legged piano-stool geometry of the iridium center. The Ir—Cl (2.434 (2) Å), Ir—P (2.312 (2) Å), and Ir—S bond lengths (2.350 (2) Å) are in the expected range. The six-membered IrPC_3_S iridacycle adopts a chair conformation. In crystals short contacts between the oxygen atoms of the water molecules and the chloride anions (O1···Cl2 3.216 (5) Å; O1···Cl2(1 - *x*, 2 - *y*, -*z*) 3.196 (6) Å) indicate according to Steiner (2002) weak O—H···Cl hydrogen bonds (H10···Cl2 2.27 Å; O1—H10···Cl2 177°; H2O···Cl2(1 - *x*, 2 - *y*, -*z*) 2.26 Å; O1—H2O···Cl2 165°).

### Experimental

To a methanol solution (30 ml) of [{IrCl_2_(η^5^-C_5_Me_5_)}_2_] (0.10 g, 0.16 mmol) Ph_2_P(CH_2_)_3_SPh (0.32 mmol) was added with stirring and then the solution was heated under reflux for three hours. The reaction mixture was cooled down to room temperature, water (2 ml) was added and after storage in a freezer at -70°C overnight the precipitate obtained was filtered off, washed with ether (3 × 2 ml), and dried in vacuum. Crystals of the title compound suitable for X-ray diffraction analysis were obtained from acetone solution at room temperature.

### Refinement

The H atoms, except those from the water molecules, were placed in calculated positions and constrained to ride on their parent atoms. Hydrogen atoms of the water molecules were found in the difference Fourier map and refined using the restraints "*DFIX* 0.960 0.020 H2O O1" and "*DFIX* 0.960 0.020 O1 H10". To modulate the anisotropic displacement parameters of the ellipsoids from the pentamethylcyclopentadienyl ligand the restraints "DELU 0.010 0.010 C1 C2 C3 C4 C5 C6 C7 C8 C9 C10", "SIMU 0.040 0.080 1.700 C1 C2 C3 C4 C5 C6 C7 C8 C9 C10" and "ISOR 0.100 C1 C2 C3 C4 C5 C6 C7 C8 C9 C10" were used. The H atoms on C6 have been refined disordered over two equally occupied positions.

### Crystal data

**Table:**

[Ir(C_10_H_15_)Cl(C_21_H_21_PS)]Cl·H_2_O	*F*(000) = 1496
*M**_r_* = 752.74	*D*_x_ = 1.671 Mg m^−^^3^
Monoclinic, *P*2_1_/*n*	Mo *K*α radiation, λ = 0.71073 Å
Hall symbol: -P 2yn	Cell parameters from 14059 reflections
*a* = 11.0720 (3) Å	θ = 2.9–28.1°
*b* = 8.9617 (2) Å	µ = 4.79 mm^−^^1^
*c* = 30.6266 (7) Å	*T* = 100 K
β = 100.000 (2)°	Block, yellow
*V* = 2992.72 (13) Å^3^	0.25 × 0.2 × 0.2 mm
*Z* = 4	

### Data collection

**Table:**

Oxford Diffraction Gemini S diffractometer	5098 reflections with *I* > 2σ(*I*)
Radiation source: fine-focus sealed tube	*R*_int_ = 0.047
Graphite monochromator	θ_max_ = 25.5°, θ_min_ = 2.9°
ω scans	*h* = −13→13
Absorption correction: multi-scan (*CrysAlis PRO*; Oxford Diffraction, 2006)	*k* = −10→10
*T*_min_ = 0.638, *T*_max_ = 1.000	*l* = −35→37
27405 measured reflections	2 standard reflections every 50 reflections
5513 independent reflections	intensity decay: none

### Refinement

**Table:**

Refinement on *F*^2^	Primary atom site location: structure-invariant direct methods
Least-squares matrix: full	Secondary atom site location: difference Fourier map
*R*[*F*^2^ > 2σ(*F*^2^)] = 0.041	Hydrogen site location: inferred from neighbouring sites
*wR*(*F*^2^) = 0.088	H atoms treated by a mixture of independent and constrained refinement
*S* = 1.25	*w* = 1/[σ^2^(*F*_o_^2^) + (0.*P*)^2^ + 29.4455*P*] where *P* = (*F*_o_^2^ + 2*F*_c_^2^)/3
5513 reflections	(Δ/σ)_max_ = 0.001
337 parameters	Δρ_max_ = 1.68 e Å^−^^3^
147 restraints	Δρ_min_ = −1.50 e Å^−^^3^

### Special details

**Table:**

Geometry. All e.s.d.'s (except the e.s.d. in the dihedral angle between two l.s. planes) are estimated using the full covariance matrix. The cell e.s.d.'s are taken into account individually in the estimation of e.s.d.'s in distances, angles and torsion angles; correlations between e.s.d.'s in cell parameters are only used when they are defined by crystal symmetry. An approximate (isotropic) treatment of cell e.s.d.'s is used for estimating e.s.d.'s involving l.s. planes.
Refinement. Refinement of *F*^2^ against ALL reflections. The weighted *R*-factor *wR* and goodness of fit *S* are based on *F*^2^, conventional *R*-factors *R* are based on *F*, with *F* set to zero for negative *F*^2^. The threshold expression of *F*^2^ > σ(*F*^2^) is used only for calculating *R*-factors(gt) *etc*. and is not relevant to the choice of reflections for refinement. *R*-factors based on *F*^2^ are statistically about twice as large as those based on *F*, and *R*- factors based on ALL data will be even larger.

### Fractional atomic coordinates and isotropic or equivalent isotropic displacement parameters (Å^2^)

**Table:**

	*x*	*y*	*z*	*U*_iso_*/*U*_eq_	Occ. (<1)
C1	0.4071 (6)	0.1087 (7)	0.1078 (2)	0.0180 (13)	
C2	0.5278 (6)	0.1774 (8)	0.1208 (2)	0.0196 (13)	
C3	0.5326 (6)	0.3021 (7)	0.0928 (2)	0.0183 (13)	
C4	0.4139 (6)	0.3191 (7)	0.0648 (2)	0.0175 (13)	
C5	0.3395 (6)	0.1940 (7)	0.0729 (2)	0.0192 (13)	
C6	0.3704 (6)	−0.0502 (7)	0.1281 (2)	0.0191 (8)	
H6A	0.4355	−0.0819	0.1512	0.029*	0.50
H6B	0.3577	−0.1239	0.1051	0.029*	0.50
H6C	0.2964	−0.0382	0.1401	0.029*	0.50
H6D	0.2909	−0.0808	0.1131	0.029*	0.50
H6E	0.3687	−0.0388	0.1592	0.029*	0.50
H6F	0.4300	−0.1245	0.1241	0.029*	0.50
C7	0.6279 (6)	0.1159 (7)	0.1545 (2)	0.0191 (8)	
H7A	0.6561	0.0236	0.1440	0.029*	
H7B	0.5977	0.0986	0.1816	0.029*	
H7C	0.6945	0.1859	0.1598	0.029*	
C8	0.6406 (6)	0.3992 (8)	0.0898 (3)	0.0263 (16)	
H8A	0.7006	0.3884	0.1163	0.039*	
H8B	0.6146	0.5014	0.0867	0.039*	
H8C	0.6758	0.3702	0.0646	0.039*	
C9	0.3851 (6)	0.4222 (7)	0.0260 (2)	0.0191 (8)	
H9A	0.4098	0.3770	0.0005	0.029*	
H9B	0.4284	0.5144	0.0327	0.029*	
H9C	0.2985	0.4413	0.0199	0.029*	
C10	0.2196 (6)	0.1528 (8)	0.0457 (2)	0.0249 (15)	
H10A	0.2331	0.1003	0.0197	0.037*	
H10B	0.1729	0.2416	0.0371	0.037*	
H10C	0.1752	0.0900	0.0628	0.037*	
C11	0.6084 (6)	0.5606 (7)	0.1848 (2)	0.0197 (14)	
C12	0.6887 (6)	0.6611 (8)	0.1706 (2)	0.0238 (15)	
H12	0.6604	0.7311	0.1488	0.029*	
C13	0.8121 (6)	0.6555 (9)	0.1895 (2)	0.0273 (16)	
H13	0.8668	0.7219	0.1801	0.033*	
C14	0.8542 (6)	0.5532 (8)	0.2220 (2)	0.0253 (16)	
H14	0.9367	0.5517	0.2348	0.030*	
C15	0.7734 (7)	0.4523 (8)	0.2356 (2)	0.0275 (17)	
H15	0.8021	0.3819	0.2572	0.033*	
C16	0.6500 (6)	0.4559 (8)	0.2171 (2)	0.0247 (16)	
H16	0.5957	0.3886	0.2263	0.030*	
C17	0.3816 (6)	0.6217 (8)	0.2063 (2)	0.0219 (15)	
H17A	0.3965	0.5426	0.2282	0.026*	
H17B	0.4207	0.7115	0.2197	0.026*	
C18	0.2441 (6)	0.6484 (8)	0.1944 (2)	0.0223 (15)	
H18A	0.2282	0.7100	0.1680	0.027*	
H18B	0.2177	0.7039	0.2182	0.027*	
C19	0.1674 (6)	0.5070 (8)	0.1861 (2)	0.0215 (14)	
H19A	0.0819	0.5325	0.1853	0.026*	
H19B	0.1914	0.4392	0.2107	0.026*	
C20	0.1165 (6)	0.5512 (7)	0.0942 (2)	0.0190 (14)	
C21	0.1912 (6)	0.6303 (7)	0.0707 (2)	0.0200 (15)	
H21	0.2737	0.6051	0.0737	0.024*	
C22	0.1455 (7)	0.7462 (8)	0.0428 (2)	0.0243 (16)	
H22	0.1964	0.7978	0.0268	0.029*	
C23	0.0225 (7)	0.7842 (8)	0.0389 (2)	0.0269 (17)	
H23	−0.0089	0.8620	0.0203	0.032*	
C24	−0.0543 (6)	0.7077 (7)	0.0626 (3)	0.0256 (17)	
H24	−0.1363	0.7347	0.0600	0.031*	
C25	−0.0078 (6)	0.5913 (8)	0.0899 (2)	0.0209 (15)	
H25	−0.0590	0.5391	0.1056	0.025*	
C26	0.0716 (6)	0.2595 (8)	0.1303 (2)	0.0201 (14)	
C27	0.0810 (6)	0.1559 (9)	0.1647 (2)	0.0269 (16)	
H27	0.1415	0.1680	0.1896	0.032*	
C28	0.0021 (7)	0.0352 (8)	0.1624 (3)	0.0300 (18)	
H28	0.0100	−0.0335	0.1854	0.036*	
C29	−0.0886 (6)	0.0174 (8)	0.1256 (3)	0.0279 (17)	
H29	−0.1429	−0.0624	0.1243	0.034*	
C30	−0.0991 (6)	0.1177 (8)	0.0907 (3)	0.0256 (16)	
H30	−0.1592	0.1042	0.0658	0.031*	
C31	−0.0201 (6)	0.2374 (8)	0.0931 (2)	0.0223 (15)	
H31	−0.0278	0.3047	0.0697	0.027*	
P1	0.18229 (15)	0.41058 (19)	0.13450 (6)	0.0167 (4)	
S1	0.45073 (15)	0.57108 (18)	0.15886 (5)	0.0171 (3)	
Cl1	0.38749 (14)	0.24277 (19)	0.20829 (5)	0.0202 (3)	
Ir1	0.38099 (2)	0.33401 (3)	0.133078 (8)	0.01399 (8)	
O1	0.3710 (5)	0.8621 (6)	−0.00804 (19)	0.0332 (13)	
Cl2	0.61713 (16)	0.8410 (2)	0.06493 (6)	0.0287 (4)	
H1O	0.442 (5)	0.853 (10)	0.014 (2)	0.05 (3)*	
H2O	0.376 (14)	0.960 (7)	−0.020 (5)	0.15 (6)*	

### Atomic displacement parameters (Å^2^)

**Table:**

	*U*^11^	*U*^22^	*U*^33^	*U*^12^	*U*^13^	*U*^23^
C1	0.020 (3)	0.012 (3)	0.022 (4)	0.002 (2)	0.004 (3)	−0.008 (2)
C2	0.022 (3)	0.016 (3)	0.021 (3)	0.007 (3)	0.007 (2)	−0.005 (3)
C3	0.018 (3)	0.019 (3)	0.021 (3)	0.000 (2)	0.013 (2)	−0.006 (2)
C4	0.023 (3)	0.015 (3)	0.016 (3)	0.005 (3)	0.008 (2)	−0.004 (2)
C5	0.023 (3)	0.015 (3)	0.020 (3)	0.003 (3)	0.006 (3)	−0.007 (2)
C6	0.021 (2)	0.0149 (18)	0.023 (2)	−0.0029 (15)	0.0073 (16)	−0.0067 (15)
C7	0.021 (2)	0.0149 (18)	0.023 (2)	−0.0029 (15)	0.0073 (16)	−0.0067 (15)
C8	0.025 (4)	0.016 (3)	0.041 (5)	−0.004 (3)	0.016 (3)	−0.007 (3)
C9	0.021 (2)	0.0149 (18)	0.023 (2)	−0.0029 (15)	0.0073 (16)	−0.0067 (15)
C10	0.024 (3)	0.025 (4)	0.024 (4)	0.003 (3)	0.001 (3)	−0.008 (3)
C11	0.021 (4)	0.019 (3)	0.018 (4)	−0.004 (3)	0.002 (3)	−0.006 (3)
C12	0.028 (4)	0.020 (3)	0.024 (4)	−0.004 (3)	0.006 (3)	−0.004 (3)
C13	0.026 (4)	0.027 (4)	0.030 (4)	−0.012 (3)	0.010 (3)	−0.006 (3)
C14	0.019 (4)	0.035 (4)	0.021 (4)	−0.008 (3)	0.002 (3)	−0.005 (3)
C15	0.027 (4)	0.029 (4)	0.025 (4)	−0.003 (3)	−0.001 (3)	0.002 (3)
C16	0.023 (4)	0.025 (4)	0.026 (4)	−0.006 (3)	0.002 (3)	0.005 (3)
C17	0.022 (4)	0.031 (4)	0.014 (4)	−0.003 (3)	0.007 (3)	−0.008 (3)
C18	0.028 (4)	0.021 (4)	0.020 (4)	0.000 (3)	0.011 (3)	−0.005 (3)
C19	0.022 (3)	0.023 (4)	0.020 (4)	0.003 (3)	0.007 (3)	0.005 (3)
C20	0.021 (3)	0.015 (3)	0.020 (4)	0.002 (3)	0.000 (3)	−0.002 (3)
C21	0.023 (4)	0.015 (3)	0.022 (4)	−0.001 (3)	0.003 (3)	−0.004 (3)
C22	0.030 (4)	0.019 (4)	0.023 (4)	0.002 (3)	0.002 (3)	0.002 (3)
C23	0.028 (4)	0.021 (4)	0.030 (4)	0.003 (3)	0.000 (3)	0.000 (3)
C24	0.016 (3)	0.018 (4)	0.040 (5)	0.006 (3)	−0.003 (3)	−0.005 (3)
C25	0.016 (3)	0.023 (4)	0.024 (4)	0.008 (3)	0.004 (3)	−0.003 (3)
C26	0.013 (3)	0.024 (4)	0.025 (4)	−0.001 (3)	0.007 (3)	0.000 (3)
C27	0.021 (4)	0.029 (4)	0.030 (4)	−0.006 (3)	0.004 (3)	0.005 (3)
C28	0.027 (4)	0.022 (4)	0.041 (5)	−0.005 (3)	0.006 (3)	0.005 (3)
C29	0.022 (4)	0.024 (4)	0.039 (5)	−0.006 (3)	0.010 (3)	−0.002 (3)
C30	0.017 (3)	0.026 (4)	0.033 (4)	0.000 (3)	0.004 (3)	−0.008 (3)
C31	0.017 (3)	0.022 (4)	0.028 (4)	0.004 (3)	0.005 (3)	−0.003 (3)
P1	0.0149 (8)	0.0171 (8)	0.0187 (9)	0.0000 (7)	0.0049 (7)	0.0000 (7)
S1	0.0191 (8)	0.0135 (8)	0.0187 (8)	−0.0004 (6)	0.0035 (6)	−0.0015 (6)
Cl1	0.0190 (8)	0.0230 (8)	0.0187 (9)	−0.0003 (7)	0.0037 (6)	0.0050 (7)
Ir1	0.01420 (13)	0.01298 (13)	0.01507 (13)	0.00024 (10)	0.00329 (9)	0.00059 (10)
O1	0.029 (3)	0.031 (3)	0.038 (3)	−0.009 (2)	0.001 (2)	−0.002 (3)
Cl2	0.0347 (10)	0.0268 (9)	0.0233 (9)	0.0056 (8)	0.0014 (7)	−0.0008 (8)

### Geometric parameters (Å, º)

**Table:**

C1—C5	1.419 (9)	C15—C16	1.386 (10)
C1—C2	1.462 (9)	C15—H15	0.9300
C1—C6	1.634 (9)	C16—H16	0.9300
C1—Ir1	2.199 (6)	C17—C18	1.521 (9)
C2—C3	1.414 (9)	C17—S1	1.815 (7)
C2—C7	1.484 (9)	C17—H17A	0.9700
C2—Ir1	2.228 (6)	C17—H17B	0.9700
C3—C4	1.448 (9)	C18—C19	1.523 (9)
C3—C8	1.495 (9)	C18—H18A	0.9700
C3—Ir1	2.266 (6)	C18—H18B	0.9700
C4—C5	1.438 (9)	C19—P1	1.834 (7)
C4—C9	1.494 (9)	C19—H19A	0.9700
C4—Ir1	2.189 (6)	C19—H19B	0.9700
C5—C10	1.486 (9)	C20—C21	1.383 (10)
C5—Ir1	2.212 (6)	C20—C25	1.407 (9)
C6—H6A	0.9600	C20—P1	1.824 (7)
C6—H6B	0.9600	C21—C22	1.384 (9)
C6—H6C	0.9600	C21—H21	0.9300
C6—H6D	0.9600	C22—C23	1.389 (10)
C6—H6E	0.9600	C22—H22	0.9300
C6—H6F	0.9600	C23—C24	1.391 (10)
C7—H7A	0.9600	C23—H23	0.9300
C7—H7B	0.9600	C24—C25	1.380 (10)
C7—H7C	0.9600	C24—H24	0.9300
C8—H8A	0.9600	C25—H25	0.9300
C8—H8B	0.9600	C26—C27	1.393 (10)
C8—H8C	0.9600	C26—C31	1.403 (10)
C9—H9A	0.9600	C26—P1	1.816 (7)
C9—H9B	0.9600	C27—C28	1.385 (10)
C9—H9C	0.9600	C27—H27	0.9300
C10—H10A	0.9600	C28—C29	1.381 (11)
C10—H10B	0.9600	C28—H28	0.9300
C10—H10C	0.9600	C29—C30	1.385 (11)
C11—C16	1.383 (10)	C29—H29	0.9300
C11—C12	1.388 (10)	C30—C31	1.378 (10)
C11—S1	1.790 (7)	C30—H30	0.9300
C12—C13	1.390 (10)	C31—H31	0.9300
C12—H12	0.9300	P1—Ir1	2.3121 (17)
C13—C14	1.372 (11)	S1—Ir1	2.3498 (16)
C13—H13	0.9300	Cl1—Ir1	2.4337 (16)
C14—C15	1.387 (10)	O1—H1O	0.95 (2)
C14—H14	0.9300	O1—H2O	0.96 (2)
			
C5—C1—C2	108.4 (6)	C18—C17—S1	113.0 (5)
C5—C1—C6	128.6 (6)	C18—C17—H17A	109.0
C2—C1—C6	122.7 (6)	S1—C17—H17A	109.0
C5—C1—Ir1	71.7 (4)	C18—C17—H17B	109.0
C2—C1—Ir1	71.8 (3)	S1—C17—H17B	109.0
C6—C1—Ir1	127.6 (4)	H17A—C17—H17B	107.8
C3—C2—C1	107.3 (6)	C17—C18—C19	114.5 (6)
C3—C2—C7	127.8 (6)	C17—C18—H18A	108.6
C1—C2—C7	124.7 (6)	C19—C18—H18A	108.6
C3—C2—Ir1	73.1 (4)	C17—C18—H18B	108.6
C1—C2—Ir1	69.6 (3)	C19—C18—H18B	108.6
C7—C2—Ir1	126.5 (5)	H18A—C18—H18B	107.6
C2—C3—C4	108.5 (6)	C18—C19—P1	113.9 (5)
C2—C3—C8	127.7 (6)	C18—C19—H19A	108.8
C4—C3—C8	123.7 (6)	P1—C19—H19A	108.8
C2—C3—Ir1	70.2 (4)	C18—C19—H19B	108.8
C4—C3—Ir1	68.2 (3)	P1—C19—H19B	108.8
C8—C3—Ir1	129.5 (5)	H19A—C19—H19B	107.7
C5—C4—C3	107.6 (6)	C21—C20—C25	118.9 (6)
C5—C4—C9	124.7 (6)	C21—C20—P1	120.1 (5)
C3—C4—C9	125.7 (6)	C25—C20—P1	120.6 (5)
C5—C4—Ir1	71.8 (4)	C20—C21—C22	121.3 (6)
C3—C4—Ir1	73.9 (4)	C20—C21—H21	119.3
C9—C4—Ir1	132.3 (4)	C22—C21—H21	119.3
C1—C5—C4	107.9 (6)	C21—C22—C23	118.9 (7)
C1—C5—C10	126.3 (6)	C21—C22—H22	120.5
C4—C5—C10	125.5 (6)	C23—C22—H22	120.5
C1—C5—Ir1	70.7 (4)	C22—C23—C24	121.0 (7)
C4—C5—Ir1	70.1 (4)	C22—C23—H23	119.5
C10—C5—Ir1	130.0 (5)	C24—C23—H23	119.5
C1—C6—H6A	109.5	C25—C24—C23	119.4 (6)
C1—C6—H6B	109.5	C25—C24—H24	120.3
H6A—C6—H6B	109.5	C23—C24—H24	120.3
C1—C6—H6C	109.5	C24—C25—C20	120.4 (7)
H6A—C6—H6C	109.5	C24—C25—H25	119.8
H6B—C6—H6C	109.5	C20—C25—H25	119.8
C1—C6—H6D	109.5	C27—C26—C31	117.9 (6)
H6A—C6—H6D	141.1	C27—C26—P1	118.9 (5)
H6B—C6—H6D	56.3	C31—C26—P1	123.2 (5)
H6C—C6—H6D	56.3	C28—C27—C26	121.2 (7)
C1—C6—H6E	109.5	C28—C27—H27	119.4
H6A—C6—H6E	56.3	C26—C27—H27	119.4
H6B—C6—H6E	141.1	C29—C28—C27	119.7 (7)
H6C—C6—H6E	56.3	C29—C28—H28	120.2
H6D—C6—H6E	109.5	C27—C28—H28	120.2
C1—C6—H6F	109.5	C28—C29—C30	120.3 (7)
H6A—C6—H6F	56.3	C28—C29—H29	119.8
H6B—C6—H6F	56.3	C30—C29—H29	119.8
H6C—C6—H6F	141.1	C31—C30—C29	119.8 (7)
H6D—C6—H6F	109.5	C31—C30—H30	120.1
H6E—C6—H6F	109.5	C29—C30—H30	120.1
C2—C7—H7A	109.5	C30—C31—C26	121.1 (7)
C2—C7—H7B	109.5	C30—C31—H31	119.5
H7A—C7—H7B	109.5	C26—C31—H31	119.5
C2—C7—H7C	109.5	C26—P1—C20	106.4 (3)
H7A—C7—H7C	109.5	C26—P1—C19	104.6 (3)
H7B—C7—H7C	109.5	C20—P1—C19	99.9 (3)
C3—C8—H8A	109.5	C26—P1—Ir1	114.2 (2)
C3—C8—H8B	109.5	C20—P1—Ir1	117.5 (2)
H8A—C8—H8B	109.5	C19—P1—Ir1	112.6 (2)
C3—C8—H8C	109.5	C11—S1—C17	99.9 (3)
H8A—C8—H8C	109.5	C11—S1—Ir1	109.9 (2)
H8B—C8—H8C	109.5	C17—S1—Ir1	109.6 (2)
C4—C9—H9A	109.5	C4—Ir1—C1	63.5 (2)
C4—C9—H9B	109.5	C4—Ir1—C5	38.1 (2)
H9A—C9—H9B	109.5	C1—Ir1—C5	37.5 (2)
C4—C9—H9C	109.5	C4—Ir1—C2	63.4 (2)
H9A—C9—H9C	109.5	C1—Ir1—C2	38.5 (2)
H9B—C9—H9C	109.5	C5—Ir1—C2	63.5 (2)
C5—C10—H10A	109.5	C4—Ir1—C3	37.9 (2)
C5—C10—H10B	109.5	C1—Ir1—C3	62.5 (2)
H10A—C10—H10B	109.5	C5—Ir1—C3	62.7 (2)
C5—C10—H10C	109.5	C2—Ir1—C3	36.7 (2)
H10A—C10—H10C	109.5	C4—Ir1—P1	110.65 (17)
H10B—C10—H10C	109.5	C1—Ir1—P1	117.57 (18)
C16—C11—C12	120.8 (6)	C5—Ir1—P1	97.09 (17)
C16—C11—S1	122.1 (5)	C2—Ir1—P1	156.11 (18)
C12—C11—S1	117.1 (5)	C3—Ir1—P1	147.63 (18)
C11—C12—C13	118.9 (7)	C4—Ir1—S1	106.07 (18)
C11—C12—H12	120.6	C1—Ir1—S1	153.20 (18)
C13—C12—H12	120.6	C5—Ir1—S1	143.28 (18)
C14—C13—C12	120.8 (7)	C2—Ir1—S1	114.81 (18)
C14—C13—H13	119.6	C3—Ir1—S1	93.77 (17)
C12—C13—H13	119.6	P1—Ir1—S1	89.06 (6)
C13—C14—C15	119.8 (7)	C4—Ir1—Cl1	154.38 (17)
C13—C14—H14	120.1	C1—Ir1—Cl1	92.31 (19)
C15—C14—H14	120.1	C5—Ir1—Cl1	124.50 (18)
C16—C15—C14	120.2 (7)	C2—Ir1—Cl1	92.63 (18)
C16—C15—H15	119.9	C3—Ir1—Cl1	124.70 (18)
C14—C15—H15	119.9	P1—Ir1—Cl1	87.35 (6)
C11—C16—C15	119.5 (7)	S1—Ir1—Cl1	91.82 (6)
C11—C16—H16	120.3	H1O—O1—H2O	105 (10)
C15—C16—H16	120.3		
			
C5—C1—C2—C3	−1.1 (7)	C2—C1—Ir1—C5	117.3 (6)
C6—C1—C2—C3	172.6 (5)	C6—C1—Ir1—C5	−125.1 (7)
Ir1—C1—C2—C3	−63.9 (4)	C5—C1—Ir1—C2	−117.3 (6)
C5—C1—C2—C7	−176.3 (6)	C6—C1—Ir1—C2	117.7 (7)
C6—C1—C2—C7	−2.6 (10)	C5—C1—Ir1—C3	−80.2 (4)
Ir1—C1—C2—C7	120.9 (6)	C2—C1—Ir1—C3	37.1 (4)
C5—C1—C2—Ir1	62.8 (4)	C6—C1—Ir1—C3	154.8 (6)
C6—C1—C2—Ir1	−123.5 (6)	C5—C1—Ir1—P1	63.2 (4)
C1—C2—C3—C4	4.1 (7)	C2—C1—Ir1—P1	−179.5 (3)
C7—C2—C3—C4	179.1 (6)	C6—C1—Ir1—P1	−61.9 (6)
Ir1—C2—C3—C4	−57.5 (4)	C5—C1—Ir1—S1	−109.9 (5)
C1—C2—C3—C8	−173.2 (6)	C2—C1—Ir1—S1	7.3 (7)
C7—C2—C3—C8	1.8 (11)	C6—C1—Ir1—S1	125.0 (5)
Ir1—C2—C3—C8	125.2 (7)	C5—C1—Ir1—Cl1	151.4 (4)
C1—C2—C3—Ir1	61.6 (4)	C2—C1—Ir1—Cl1	−91.3 (4)
C7—C2—C3—Ir1	−123.4 (7)	C6—C1—Ir1—Cl1	26.4 (5)
C2—C3—C4—C5	−5.5 (7)	C1—C5—Ir1—C4	118.2 (5)
C8—C3—C4—C5	171.9 (6)	C10—C5—Ir1—C4	−120.1 (8)
Ir1—C3—C4—C5	−64.3 (4)	C4—C5—Ir1—C1	−118.2 (5)
C2—C3—C4—C9	−170.2 (6)	C10—C5—Ir1—C1	121.7 (8)
C8—C3—C4—C9	7.2 (10)	C1—C5—Ir1—C2	38.2 (4)
Ir1—C3—C4—C9	131.0 (6)	C4—C5—Ir1—C2	−80.0 (4)
C2—C3—C4—Ir1	58.8 (4)	C10—C5—Ir1—C2	159.9 (7)
C8—C3—C4—Ir1	−123.8 (6)	C1—C5—Ir1—C3	79.5 (4)
C2—C1—C5—C4	−2.3 (7)	C4—C5—Ir1—C3	−38.7 (4)
C6—C1—C5—C4	−175.6 (6)	C10—C5—Ir1—C3	−158.8 (7)
Ir1—C1—C5—C4	60.5 (4)	C1—C5—Ir1—P1	−127.1 (4)
C2—C1—C5—C10	171.1 (6)	C4—C5—Ir1—P1	114.6 (3)
C6—C1—C5—C10	−2.2 (11)	C10—C5—Ir1—P1	−5.5 (6)
Ir1—C1—C5—C10	−126.1 (7)	C1—C5—Ir1—S1	134.9 (3)
C2—C1—C5—Ir1	−62.9 (4)	C4—C5—Ir1—S1	16.6 (5)
C6—C1—C5—Ir1	123.9 (6)	C10—C5—Ir1—S1	−103.5 (6)
C3—C4—C5—C1	4.8 (7)	C1—C5—Ir1—Cl1	−35.5 (4)
C9—C4—C5—C1	169.7 (6)	C4—C5—Ir1—Cl1	−153.7 (3)
Ir1—C4—C5—C1	−60.9 (4)	C10—C5—Ir1—Cl1	86.2 (6)
C3—C4—C5—C10	−168.6 (6)	C3—C2—Ir1—C4	36.3 (4)
C9—C4—C5—C10	−3.8 (10)	C1—C2—Ir1—C4	−80.1 (4)
Ir1—C4—C5—C10	125.6 (6)	C7—C2—Ir1—C4	161.2 (7)
C3—C4—C5—Ir1	65.8 (4)	C3—C2—Ir1—C1	116.4 (6)
C9—C4—C5—Ir1	−129.4 (6)	C7—C2—Ir1—C1	−118.7 (8)
C16—C11—C12—C13	−0.3 (10)	C3—C2—Ir1—C5	79.1 (4)
S1—C11—C12—C13	−179.3 (5)	C1—C2—Ir1—C5	−37.2 (4)
C11—C12—C13—C14	−0.5 (11)	C7—C2—Ir1—C5	−156.0 (7)
C12—C13—C14—C15	1.1 (11)	C1—C2—Ir1—C3	−116.4 (6)
C13—C14—C15—C16	−1.0 (11)	C7—C2—Ir1—C3	124.9 (8)
C12—C11—C16—C15	0.4 (11)	C3—C2—Ir1—P1	117.4 (5)
S1—C11—C16—C15	179.3 (6)	C1—C2—Ir1—P1	1.0 (7)
C14—C15—C16—C11	0.3 (11)	C7—C2—Ir1—P1	−117.8 (5)
S1—C17—C18—C19	76.0 (7)	C3—C2—Ir1—S1	−60.0 (4)
C17—C18—C19—P1	−71.0 (7)	C1—C2—Ir1—S1	−176.4 (3)
C25—C20—C21—C22	−0.8 (10)	C7—C2—Ir1—S1	64.9 (6)
P1—C20—C21—C22	−174.4 (5)	C3—C2—Ir1—Cl1	−153.2 (4)
C20—C21—C22—C23	0.8 (10)	C1—C2—Ir1—Cl1	90.4 (4)
C21—C22—C23—C24	−0.1 (11)	C7—C2—Ir1—Cl1	−28.3 (6)
C22—C23—C24—C25	−0.6 (11)	C2—C3—Ir1—C4	−120.5 (5)
C23—C24—C25—C20	0.6 (10)	C8—C3—Ir1—C4	116.4 (8)
C21—C20—C25—C24	0.1 (10)	C2—C3—Ir1—C1	−39.0 (4)
P1—C20—C25—C24	173.7 (5)	C4—C3—Ir1—C1	81.4 (4)
C31—C26—C27—C28	−0.3 (11)	C8—C3—Ir1—C1	−162.1 (7)
P1—C26—C27—C28	−177.4 (6)	C2—C3—Ir1—C5	−81.5 (4)
C26—C27—C28—C29	−0.6 (12)	C4—C3—Ir1—C5	38.9 (4)
C27—C28—C29—C30	1.4 (11)	C8—C3—Ir1—C5	155.4 (7)
C28—C29—C30—C31	−1.3 (11)	C4—C3—Ir1—C2	120.5 (5)
C29—C30—C31—C26	0.3 (10)	C8—C3—Ir1—C2	−123.1 (8)
C27—C26—C31—C30	0.4 (10)	C2—C3—Ir1—P1	−137.8 (4)
P1—C26—C31—C30	177.4 (5)	C4—C3—Ir1—P1	−17.3 (5)
C27—C26—P1—C20	−163.0 (6)	C8—C3—Ir1—P1	99.1 (7)
C31—C26—P1—C20	20.1 (7)	C2—C3—Ir1—S1	128.0 (4)
C27—C26—P1—C19	−57.8 (6)	C4—C3—Ir1—S1	−111.5 (4)
C31—C26—P1—C19	125.3 (6)	C8—C3—Ir1—S1	4.9 (6)
C27—C26—P1—Ir1	65.7 (6)	C2—C3—Ir1—Cl1	33.2 (4)
C31—C26—P1—Ir1	−111.2 (6)	C4—C3—Ir1—Cl1	153.6 (3)
C21—C20—P1—C26	−142.8 (5)	C8—C3—Ir1—Cl1	−89.9 (6)
C25—C20—P1—C26	43.7 (6)	C26—P1—Ir1—C4	87.6 (3)
C21—C20—P1—C19	108.7 (6)	C20—P1—Ir1—C4	−38.0 (3)
C25—C20—P1—C19	−64.8 (6)	C19—P1—Ir1—C4	−153.3 (3)
C21—C20—P1—Ir1	−13.4 (6)	C26—P1—Ir1—C1	17.6 (3)
C25—C20—P1—Ir1	173.1 (5)	C20—P1—Ir1—C1	−108.1 (3)
C18—C19—P1—C26	−174.6 (5)	C19—P1—Ir1—C1	136.6 (3)
C18—C19—P1—C20	−64.6 (5)	C26—P1—Ir1—C5	50.8 (3)
C18—C19—P1—Ir1	60.9 (5)	C20—P1—Ir1—C5	−74.9 (3)
C16—C11—S1—C17	64.4 (6)	C19—P1—Ir1—C5	169.9 (3)
C12—C11—S1—C17	−116.6 (6)	C26—P1—Ir1—C2	16.9 (5)
C16—C11—S1—Ir1	−50.8 (6)	C20—P1—Ir1—C2	−108.8 (5)
C12—C11—S1—Ir1	128.2 (5)	C19—P1—Ir1—C2	135.9 (5)
C18—C17—S1—C11	176.7 (5)	C26—P1—Ir1—C3	98.9 (4)
C18—C17—S1—Ir1	−67.9 (5)	C20—P1—Ir1—C3	−26.7 (4)
C5—C4—Ir1—C1	36.8 (4)	C19—P1—Ir1—C3	−142.0 (4)
C3—C4—Ir1—C1	−78.4 (4)	C26—P1—Ir1—S1	−165.5 (3)
C9—C4—Ir1—C1	157.6 (7)	C20—P1—Ir1—S1	68.8 (2)
C3—C4—Ir1—C5	−115.3 (5)	C19—P1—Ir1—S1	−46.5 (2)
C9—C4—Ir1—C5	120.8 (8)	C26—P1—Ir1—Cl1	−73.7 (3)
C5—C4—Ir1—C2	80.1 (4)	C20—P1—Ir1—Cl1	160.7 (2)
C3—C4—Ir1—C2	−35.1 (4)	C19—P1—Ir1—Cl1	45.4 (2)
C9—C4—Ir1—C2	−159.1 (7)	C11—S1—Ir1—C4	−91.0 (3)
C5—C4—Ir1—C3	115.3 (5)	C17—S1—Ir1—C4	160.2 (3)
C9—C4—Ir1—C3	−124.0 (8)	C11—S1—Ir1—C1	−28.3 (5)
C5—C4—Ir1—P1	−74.6 (4)	C17—S1—Ir1—C1	−137.2 (5)
C3—C4—Ir1—P1	170.2 (3)	C11—S1—Ir1—C5	−101.6 (4)
C9—C4—Ir1—P1	46.2 (6)	C17—S1—Ir1—C5	149.6 (4)
C5—C4—Ir1—S1	−169.7 (3)	C11—S1—Ir1—C2	−23.3 (3)
C3—C4—Ir1—S1	75.0 (4)	C17—S1—Ir1—C2	−132.2 (3)
C9—C4—Ir1—S1	−49.0 (6)	C11—S1—Ir1—C3	−54.5 (3)
C5—C4—Ir1—Cl1	57.6 (6)	C17—S1—Ir1—C3	−163.4 (3)
C3—C4—Ir1—Cl1	−57.6 (6)	C11—S1—Ir1—P1	157.8 (2)
C9—C4—Ir1—Cl1	178.4 (4)	C17—S1—Ir1—P1	48.9 (2)
C5—C1—Ir1—C4	−37.4 (4)	C11—S1—Ir1—Cl1	70.5 (2)
C2—C1—Ir1—C4	79.8 (4)	C17—S1—Ir1—Cl1	−38.4 (2)
C6—C1—Ir1—C4	−162.5 (6)		

### Hydrogen-bond geometry (Å, º)

**Table:**

*D*—H···*A*	*D*—H	H···*A*	*D*···*A*	*D*—H···*A*
O1—H1*O*···Cl2	0.95 (6)	2.27 (6)	3.216 (6)	177 (10)
O1—H2*O*···Cl2^i^	0.96 (8)	2.26 (11)	3.196 (6)	165 (13)

Symmetry code: (i) −*x*+1, −*y*+2, −*z*.
